# Antidiarrheal Effect of Sechang-Zhixie-San on Acute Diarrhea Mice and Network Pharmacology Deciphering Its Characteristics and Potential Mechanisms

**DOI:** 10.1155/2020/8880298

**Published:** 2020-12-11

**Authors:** Zhiyong Li, Jianliang Li, Fengrong Zhang, Na Zhu, Zijun Sha, Dan Li, Ya Tu, Jincai Hou

**Affiliations:** ^1^Faculty of Life Sciences and Technology, Kunming University of Science and Technology, Kunming, Yunnan, China; ^2^School of Pharmacy, Minzu University of China, Beijing, China; ^3^Yunnan Province Resources for Development and Collaborative Innovation Center for New Traditional Chinese Medicine, Kunming, Yunnan, China; ^4^Institute of Chinese Materia Medica, China Academy of Chinese Medical Sciences, Beijing, China; ^5^Shandong University of Traditional Chinese Medicine, Jinan, Shandong, China; ^6^Jingjinji Lianchuang Institute of Pharmaceutical Research, Beijing, China; ^7^China Academy of Chinese Medical Sciences, Beijing, China

## Abstract

Sechang-Zhixie-San (SCZX) is an ancient prescription used for pediatric diarrhea by the Yi people in China, which consists of *Rodgersia sambucifolia* Hemsley (known as Yantuo and abbreviated as YT) and Bentonite (BN). Now, it is also a Chinese patent medicine used in the clinic to treat infantile diarrhea. Besides evaluating the antidiarrheal effect of SCZX on diarrhea mice induced by Folium Sennae, the purpose of this study is to outline the characteristics of the antidiarrheal effect and reveal the potential mechanisms of SCZX through the analysis of the mechanism and active components of YT via network pharmacology and molecular docking, combined with the research progress of BN obtained from the literature. SCZX (3.12 and 12.48 g/kg) effectively inhibited diarrhea in mice, significantly lowering the loose stool rate (LSR), loose stool level (LSL), and loose stool index (LSI). Using network pharmacology, the “herb-compound-target-pathway-pharmacological action” network was mapped to indicate the antidiarrheal mechanism of YT. And the docking results revealed that 4 components of YT including quercetin, geranyl-1-O-*α*-L-arabinopyranosyl-(1 ⟶ 6)-*β*-D-glucopyranoside, 3*α*-O-(E)-p-hydroxy-cinnamoyl-olean-12-en-27-oic acid, and daucosterol showed significant docking activities with STAT3, EGFR, and SLC10A2, involving 11 pathways such as Th17 cell differentiation, Jak-STAT signaling pathway, ErbB signaling pathway, and HIF-1 signaling pathway. According to our research results and literature reports, the antidiarrheal could be summarized into five aspects: inhibiting intestinal inflammation, acting as a barrier to the intestinal mucosal, regulating water and ion transport, involving the purification of intestinal microorganisms, and intestinal transmission, which might be dependent on multiple proteins and intervention in multiple pathways.

## 1. Introduction

Diarrhea is defined by the World Health Organization (WHO) as having three or more loose or liquid stools per day or having more stools than that is normal for that person [1]. It is usually divided into two types based on the duration: acute and chronic. Acute diarrhea is defined as <14 days in duration and persistent diarrhea episodes as ≥14 days in duration [2]. Based on the data updated by the WHO in May 2017, there were nearly 1.7 billion cases of childhood diarrheal disease each year [3]. Acute diarrhea primarily occurs in children during the first 5 years after birth, and particularly in the second half-year; in clinical practice, chronic diarrhea is also a very common gastrointestinal complaint in children. In China, chronic diarrhea is one of the most common diseases among infants and young children. Data from the WHO showed that diarrhea kills approximately 525.000 children aged <5 years each year [4]. Diarrhea is a social burden worldwide, especially in developing countries [5]. Although opiates, diphenoxylate, loperamide, adrenergic agonist, a somatostatin analog, and astringents are common therapies for nonbacterial infectious diarrhea, the current therapeutic effect is not satisfactory. Therefore, complementary and alternative medicine can be used to treat these symptoms [6].

Sechang-Zhixie-San (SCZX) originates from a secret prescription handed down by family members of Liu Guozhong, a famous doctor of Yi nationality in Yunnan Province, which has been used in China for hundreds of years [7]. Presently, it is legally used in clinical practice in China as a Chinese patent medicine. SCZX consists of Bentonite (BN) and *Rodgersia sambucifolia* Hemsley (Yantuo, YT). Bentonite, a clay mineral whose main component is montmorillonite, can be used to cleanse the digestive tract of pathogens and to protect the intestinal mucosa [8]. SCZX is often clinically used to treat autumn diarrhea, acute diarrhea, and persistent diarrhea in children [9]. SCZX has a good therapeutic effect and rapidly relieves diarrhea symptoms. However, there are no experimental studies on the effectiveness of SCZX in treating diarrhea.

The molecular targets of most traditional Chinese medicine (TCM) prescriptions and ingredients are elusive, which remain one of the biggest hurdles in the application of TCM prescription and TCM-based drug discovery [10]. Network pharmacology is based on system biology and polypharmacology [11], and molecular docking is a common approach for the highly accurate identification of interactions between receptor proteins and molecules [12]; now, these techniques offer a new approach to integrate TCM. In this study, firstly, the antidiarrheal effect of SCZX was evaluated on mice with diarrhea induced by Sennae Folium. Bentonite has been confirmed to treat diarrhea with different causative factors (virus infection, food allergy, spastic colitis, mucous colitis, and food poisoning) after oral administration [13], while YT has no clear evidence to explain the mechanism of antidiarrheal because it is rarely studied. We decided to explore the possible mechanism and material basis of YT through network pharmacology and molecular docking. Thus, combined with the research progress of BN obtained from the literature, the purpose of this study would be to outline the characteristics of the antidiarrheal effect and to reveal the potential mechanisms of SCZX.

## 2. Materials and Methods

### 2.1. Preparation of Sennae Folium

Sennae Folium was purchased from the Niu En Tang pharmacy (Anguo, China) and was identified by Prof. Ya Tu from the China Academy of Chinese Medical Sciences. The water extract of Sennae Folium was obtained according to the following protocol [14]. Sennae Folium (10 g) was steeped in 200 mL of distilled water and heated for approximately 10 min. The extract was filtered and concentrated to 0.1 g/mL (each milliliter contained dried medicinal herbs 0.1 g).

### 2.2. Care and Use of Animals

Male SPF ICR mice (body weight 20 ± 2 g) were obtained from Beijing Vital River Laboratory Animal Technology Co., Ltd. (Certificate No. SCXK(Beijing) 2016-0002; Beijing, China) and were free-fed standard chow and water under a 12/12 h dark/light cycle using adaptive feeding for 3 days. The animal protocols were approved by the Animal Care and Use Committee of the Jingjinji Lianchuang Institute of Pharmaceutical Research, China (Approval No. 020181105).

### 2.3. Drug Administration and Grouping

SCZX (lot: 17122042, Yunnan Shineway Spirin, Chuxiong, China) and loperamide hydrochloride (lot: 171205792, Xian Janssen, Xi'an, China) were prepared with distilled water. A total of 50 mice were randomized into five different treatment groups as follows: control group, model group, SCZX (3.12 g/kg and 12.48 g/kg) group, and loperamide (2.08 mg/kg) group, with 10 mice in each group. Mice in the SCZX and loperamide groups were, respectively, given SCZX and loperamide for 7 days, whereas mice in the control and model groups were given an equal amount of distilled water, 0.2 mL/10 g body weight. On the 6th and 7th day, after administration for 30 min, the mice in the model, SCZX, and loperamide groups continued to be given the Sennae Folium water extract (0.2 mL/10 g body weight) orally to construct acute diarrhea, while mice in the control group were given distilled water.

### 2.4. Determination of Antidiarrheal Effects

After administration for 7 days, the mice were restricted from food and reared individually in separate cages. The filter paper was placed underneath each cage to count loose stools, and the paper was changed once per hour. The defecation of mice was observed for 1–5 h, and the loose stools were classified into four levels based on the diameters of stain formed by loose stools on the filter paper (Table 1). Loose stool level (LSL) was defined as the calculated mean of diameters of all stool piles [15], whereas diarrhea rate (DR), loose stool rate (LSR), and loose stool index (LSI) were calculated as follows:

(1) $$\begin{array}{c} \text{DR of each group}=\frac{\text{number of diarrheic mice}}{\text{total number of mice}}\times 100\%,\\ \text{LSR of each mouse}=\frac{\text{number of loose stool}}{\text{total number of defecation}}\times 100\%,\\ \text{LSI}=\text{LSR}\times \text{LSL}. \end{array}$$

### 2.5. Small Intestinal Propulsion

After fasting (water was not restricted) for 14 h, each mouse was given orally a nutritious semisolid paste (0.5 mL each mouse) prepared with water and comprised of sodium carboxymethyl cellulose (5 g), soluble starch (4 g), whole milk powder (8 g), oral glucose (4 g), and activated carbon (2 g). After absorbing it for 20 min, all mice were sacrificed and exposed by laparotomy, and the small intestine was carefully removed to observe the leading edge of the semisolid paste. The length of the small intestine from the pylorus to ileocecal and the length of the semisolid paste promoting were measured. The formula for calculating intestinal propulsion is as follows: intestinal propulsion rate = length of semisolid paste/entire length of the small intestine.

### 2.6. Statistical Analysis

All results are expressed as means ± SD and evaluated for significance using one-way analysis of variance using SPSS17.0 (SPSS, Chicago, USA). *P* value of <0.05 was considered to have a significant difference, and *P* value <0.01 indicated an extremely significant difference.

### 2.7. Network Pharmacology

#### 2.7.1. Candidate Components Collection

Components of YT were collected from the PubChem Database (https://www.ncbi.nlm.nih.gov/pccompound/), the Chemistry Database developed by Shanghai Institute of Organic Chemistry, Chinese Academy of Science (http://202.127.145.134/scdb/default.asp), and the Traditional Chinese Medicine Systems Pharmacology Database [16] (http://lsp.nwu.edu.cn/tcmsp.php). Furthermore, we also searched the literature to supplement compounds. Chemical structures were obtained from ChemDraw software (CambridgeSoft, USA) and stored in SMILES format.

#### 2.7.2. Potential Target Prediction

The components of YT were submitted to the Swiss Target Prediction platform (http://www.swisstargetprediction.ch/) in SMILES format to predict the potential targets, and the attributes were set as “*Homo sapiens.*” Then, these targets were uploaded into the Enrichr web server (https://amp.pharm.mssm.edu/Enrichr/) to obtain gene-disease associations from DisGeNET [17]. The targets only related to diarrhea would be selected and processed by String (https://string-db.org/) to obtain protein–protein interactions (PPI); then, a network diagram (YT-target network) was constructed using Cytoscape 3.6.0.

#### 2.7.3. Building the Diarrhea-Target Database

The disease targets of diarrhea were searched in Online Mendelian Inheritance in Man (OMIM, http://omim.org/) and DrugBank (https://www.drugbank.ca/). The UniProt database was used to standardize the targets and convert them into *Homo sapiens* gene names. The targets only related to diarrhea would be selected and processed by String to obtain PPI; then, a network diagram (diarrhea-target network) was constructed using Cytoscape 3.6.0.

#### 2.7.4. Target Analysis

YT-target network was performed using MCODE to find clusters (highly interconnected regions). The common potential targets of YT and targets of diarrhea were collected, and the network topological features of these common targets in the YT-target network and diarrhea-target network were defined. These common targets were considered as the potential hub proteins for YT in the treatment of diarrhea, and the target clusters containing hub proteins were selected.

#### 2.7.5. Gene Ontology (GO) and Pathway Enrichment

The targets of diarrhea and target clusters from the YT-target network were submitted to the *Metascape* database (https://david.ncifcrf.gov) for further investigation including GO and pathway enrichment [18]. “*Homo sapiens*” was selected as the analyzed species. The threshold value was set as *P* < 0.01, minimum count 3, and enrichment factor >1.5. GO annotated and KEGG pathway analyses were also conducted.

#### 2.7.6. Network Construction and Analysis

Pathways that contained the core proteins were selected from the results of the KEGG pathway enrichment analysis and considered as the potential mechanism for YT-treating diarrhea. We collected the targets in the MCODE clusters, which can map the above pathways, and the components related to these targets were searched and determined. Thus, we conducted a “herb-compound-target-pathway” network of YT using Cytoscape 3.6.0, and the unclustered targets and their pathways would also be fitted into the visual network. The hub proteins related to YT and their covered signaling pathways would command attention and may be selected for follow-up studies.

#### 2.7.7. Absorption, Distribution, Metabolism, and Excretion (ADME) and Drug-Likeness (DL) Analysis

The properties of absorption, distribution, metabolism, and excretion are important indicators of the effectiveness of herbs and play key roles in drug discovery [19]. Computational techniques have become an alternative approach to predict ADME profiles instead of costly *in vitro* screening. The potential components of YT were submitted to AdmetSAR (http://lmmd.ecust.edu.cn/admetsar2), which was developed as a comprehensive source and open-source tool for the prediction of chemical ADME properties and drug-likeness (DL) [20]. Considering the pharmacological characteristics of the antidiarrheal [21, 22], we investigated DL, human oral bioavailability (OB), and intestinal absorption (IA), but we were not limited to these properties when identifying the potential active components of YT. According to the literature, if the component has favorable OB or IA, AdmetSAR will show “+,” attaching a probability value. AdmetSAR can provide the molecular weight, AlogP, H-bond acceptor, H-bond donor, and rotatable bonds of the compound and follow Lipinski's “rule of five” to define DL [23].

### 2.8. Molecular Docking

Molecular docking was performed between the core proteins and their associated components of YT to further explain the potential mechanism and material basis of YT in the treatment of diarrhea. The 3D structures of target proteins were downloaded from the RCSB Protein Data Bank (PDB, http://www.rcsb.org) and saved in pdb format. PyMOL (https://pymol.org) was used to isolate the macromolecules and their protoligands, and the structures of the macromolecules were then optimized using AutoDock Tools 1.5.6. It removed water molecules, added hydrogen atoms, repaired the charges by adding a Gasteiger charge, and was saved in pdbqt format. The structure information of small molecule compounds of YT was obtained from PubChem (https://pubchem.ncbi.nlm.nih.gov/) and then kept in mol2 format. AutoDockTools 1.5.6 was used to count the number of rotatable chemical bonds, and the compounds were saved in pdbqt format. The macromolecule grid box was defined according to the protoligand site using AutoDockTools 1.5.6. Furthermore, an accurate docking with the components of YT and target proteins was performed using AutoDock vina 1.1.2, setting the energy range = 5 and exhaustiveness = 100, and the best docking conformation was analyzed with PyMOL and LigPlot^+^ v.2.2.

## 3. Results

### 3.1. SCZX Reduces Diarrhea Caused by Sennae Folium in Mice

**Figure 1:**
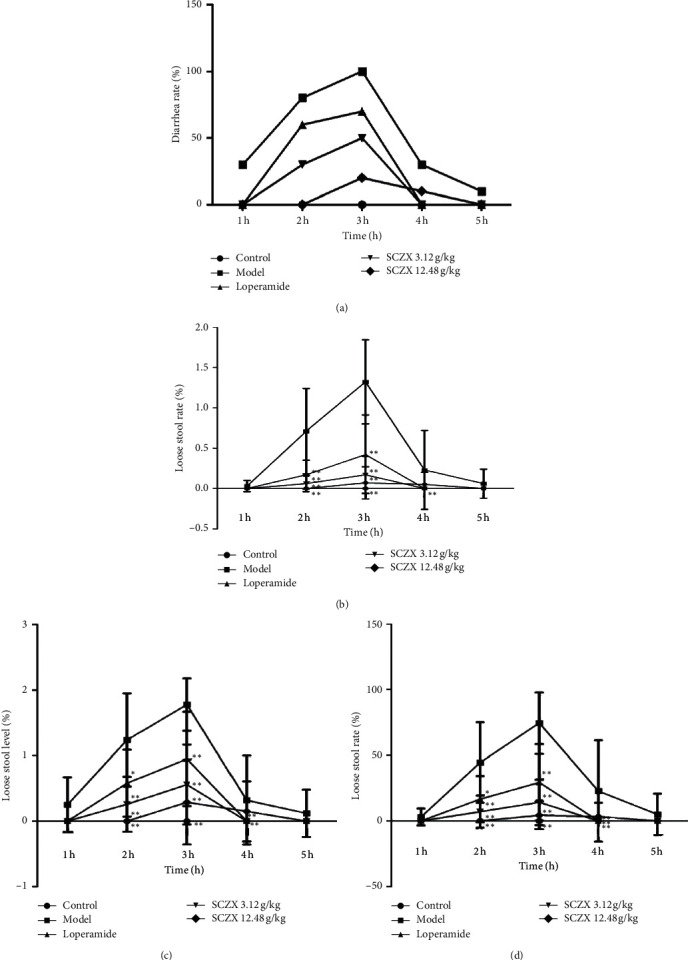
Effects of SCZX on stool parameters of mice with diarrhea induced by Sennae Folium. *Note*. Diarrhea rate (DR, %) (a); loose stool rate (LSR, %) (b); loose stool level (LSL, %) (c); loose stool index (LSI) (d); ^*∗∗*^*P* < 0.01 versus model group; *N* = 10.

Compared to the control group, Sennae Folium could increase the rate of diarrhea in mice at the second and third hours after administration; the LSR, LSL, and LSI of mice significantly increased (*P* < 0.01), while only LSR was significantly different at the 4th h (*P* < 0.01). Compared to the model group, the DR of mice treated with SCZX (3.12 g/kg and 12.48 g/kg) and loperamide decreased at all time points. At the second and third hours after administration, the DR, LSR, LSL, and LSI of mice in SCZX groups significantly decreased (*P* < 0.01). The results are shown in Figure 1.

### 3.2. SCZX Inhibits Small Bowel Movement in Mice with Diarrhea

**Figure 2:**
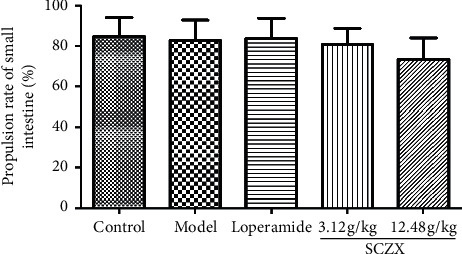
Effects of SCZX on small intestine propulsion rate in mice with diarrhea (*N* = 10).

Compared with the model group, the small intestine propulsion rate of mice in SCZX groups slightly decreased but did not reach statistical significance (Figure 2). This suggested that SCZX might possess a certain inhibitory effect on small intestine propulsion in mice with diarrhea.

### 3.3. Network Pharmacology Analysis for YT

#### 3.3.1. Target Prediction of YT-Treating Diarrhea

**Figure 3:**
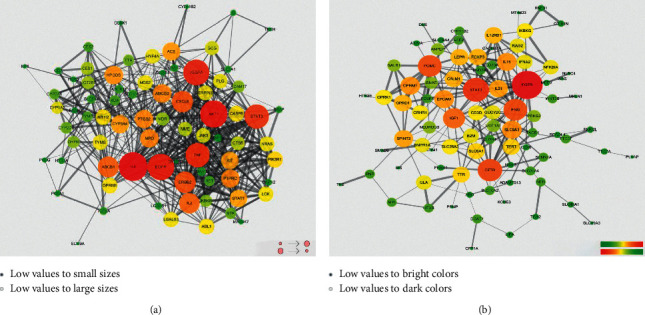
PPI network of YT and diarrhea. *Note*. Potential targets and PPI network of YT in treating diarrhea (a); PPI network of diarrhea (b). PPI: protein and protein interaction. Circular node size represents the degree value, and the redder a node is, the larger its degree value is. The edges represent the relationships between nodes, and the thicker the edge, the larger the combined score value.

A total of 48 chemical constituents of YT were collected. There were 3,985 predicted targets obtained via the *SwissTarget* Prediction platform, and after eliminating duplicates, only 856 targets were left. Based on the analysis of gene–disease associations from DisGeNET, there were 92 targets related to diarrhea, severe diarrhea, chronic diarrhea, diarrheal disorder, with tufting enteropathy, congenital intermittent diarrhea, insulin-dependent diabetes mellitus secretory diarrhea syndrome, hemorrhagic diarrhea, secretory diarrhea, and antibiotic-associated diarrhea. Only 79 targets were obtained after eliminating the duplicates, which were considered as the potential targets for YT to treat diarrhea (Figure 3).

#### 3.3.2. Definition of Hub Targets for YT

**Figure 4:**
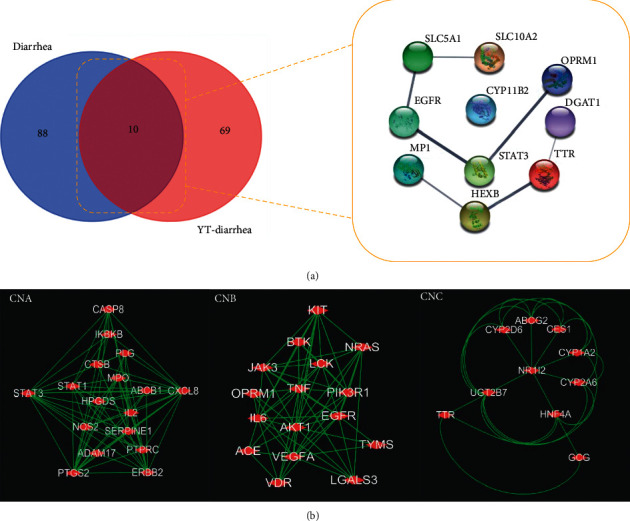
Hub proteins and cluster networks of YT. *Note*. Interactive core targets of YT in the treatment of diarrhea (a); significant modules in PPI network of YT (b).

There are 98 targets connected with diarrhea, which were collected from the DrugBank and OMIM, and the PPI network is shown in Figure 3. The 10 common targets in the Venn Diagram, which might display the intersection of targets of YT and diarrhea, were considered as the core proteins for YT in the treatment of diarrhea. We obtained three cluster networks depending on these 79 targets related to YT via MCODE, and the topological features were shown in Table S1. Only two cluster networks contained the core proteins of YT, and they were selected for further analysis and defined as cluster networks A and B (CNA and CNB). These results were shown in Figure 4.

#### 3.3.3. GO and KEGG Pathway Analysis

**Figure 5:**
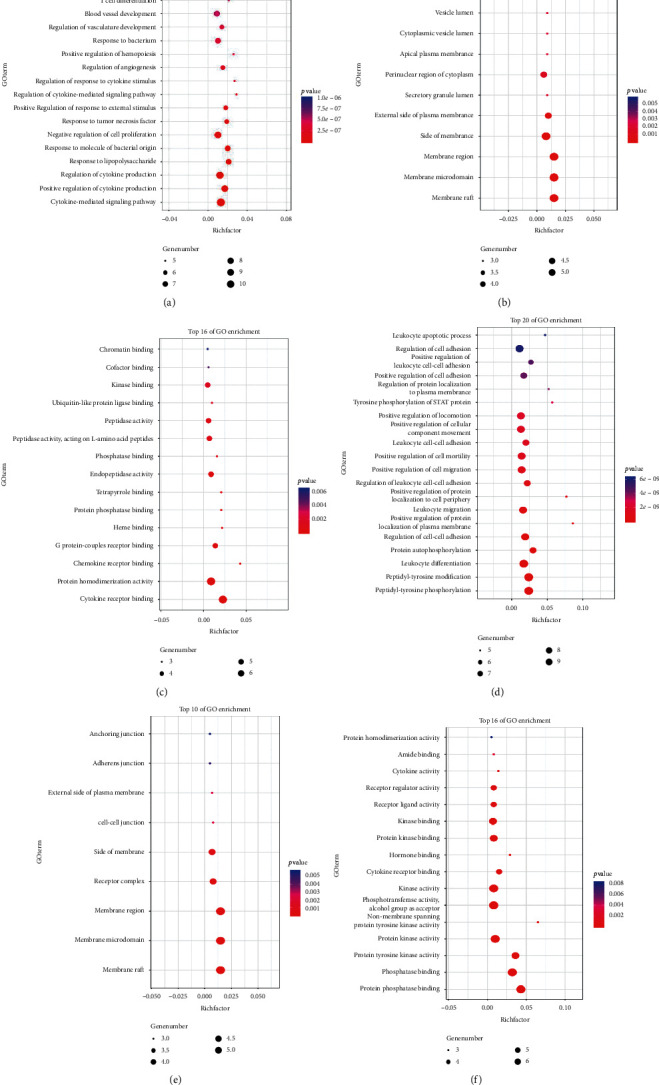
Gene ontology enrichment of cluster networks A and B. *Note*. Biological process of CNA (a); cellular component of CNA (b); molecular function of CNA (c); biological process of CNB (d); cellular component of CNB (e); molecular function of CNB (f).

Metascape is a web-based portal designed to provide a comprehensive gene list annotation and analysis resource, which combines functional enrichment, interactome analysis, gene annotation, and membership search to leverage over 40 independent knowledge bases within an integrated portal. Metascape is an effective and efficient tool for experimental biologists to comprehensively analyze and interpret OMICs-based studies in the big data era [18]. The results of the *Metascape* enrichment analysis are listed from low to high according to the -lgP values. GO enrichment analysis comprised three parts: biological process (BP), cellular component (CC), and molecular function (MF), and the BP, CC, and MF of CNA and CNB from the YT-target network are shown in Figure 5.

**Figure 6:**
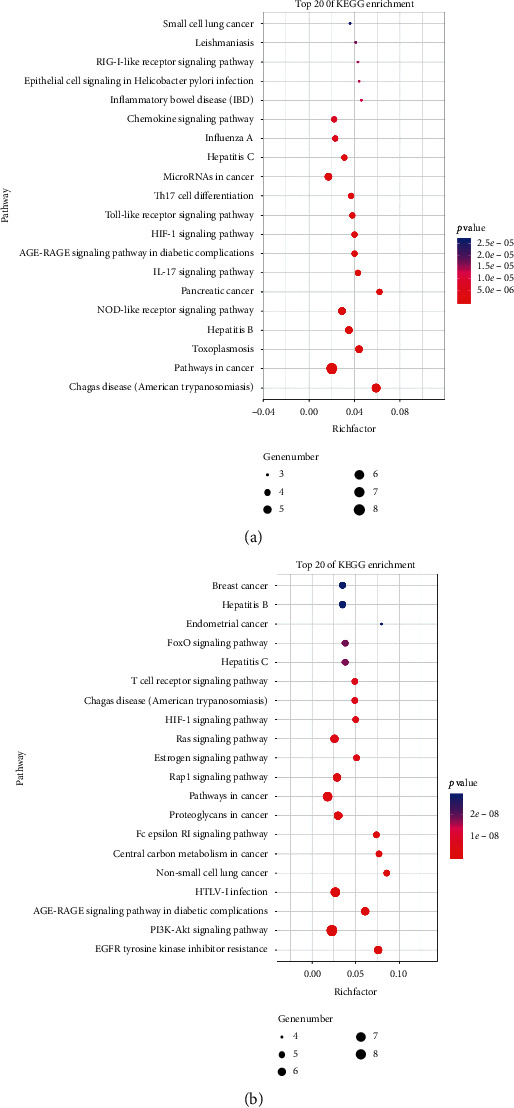
KEGG enrichment of CNA and CNB.

KEGG pathway enrichment analysis of CNA and CNB was conducted using Metascape, and the top 20 pathways are shown in Figure 6. The unclustered targets of YT-treating diarrhea were also enriched to obtain their signaling pathways. Among them, the pathways related to diarrhea and containing the core proteins of YT are listed in Table 2. Only five proteins, including STAT3, EGFR, SLC5A1, SLC10A2, and OPRM1, were found to map to the abovementioned signaling pathways.

#### 3.3.4. Network Construction of YT in the Treatment of Diarrhea

**Figure 7:**
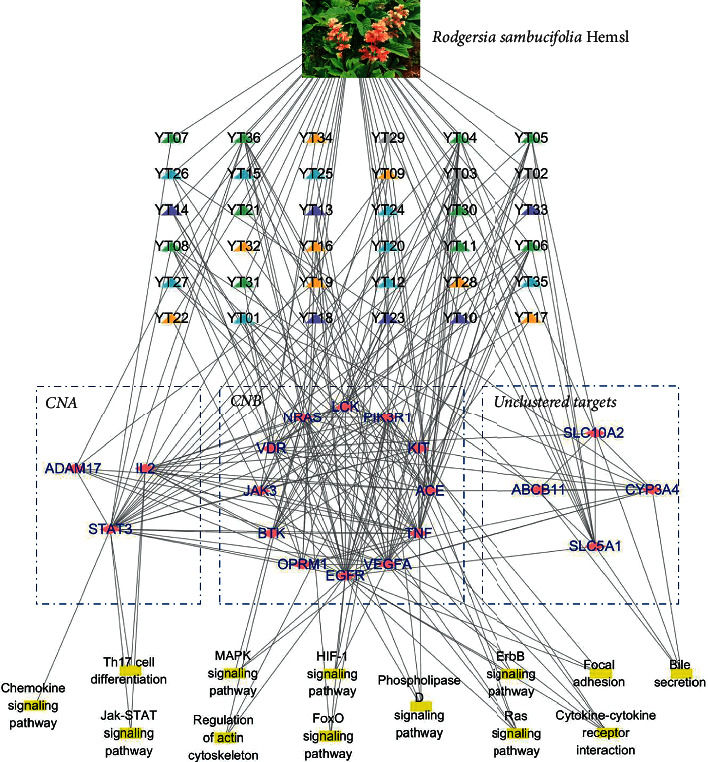
The “herb-component-target-pathway” network of YT-treating diarrhea. *Note*. The triangle node represents the component of YT. The orange node represents the component with favorable human OB, IA, and DL; the blue node represents the component with DL and favorable human OB or IA; the green node represents the component with only DL properties; the violet node represents the component with human OB or IA; the gray node represents the component without any of the above properties. The pink diamond node represents the target protein and the yellow rectangle node represents the pathway.

The pathways in Table 2, the covering targets in CNA and CNB, and the unclustered targets were collected. The 36 components of YT were also selected, which were associated with these targets. To investigate the therapeutic mechanism for the treatment of diarrhea, a “herb-compound-target-pathway” network of YT was constructed using Cytoscape 3.6.0 (Figure 7). The DL, human OB, and IA of these components were screened, and these components, which were closely related to STAT3, EGFR, SLC5A1, SLC10A2, and OPRM1, received special attention. The results are shown in Table 3. Among them, anethole (YT09), connecting with STAT3 and EGFR; 3, 4-dihydroxybenzoic acid (YT16) relating to EGFR and OPRM1; and benzene, 1, 2-dimethoxy-4-(1-propenyl)- (YT17) targeting to EGFR were predicted as having favorable DL, human OB, and IA.

### 3.4. Molecular Docking Analysis

**Figure 8:**
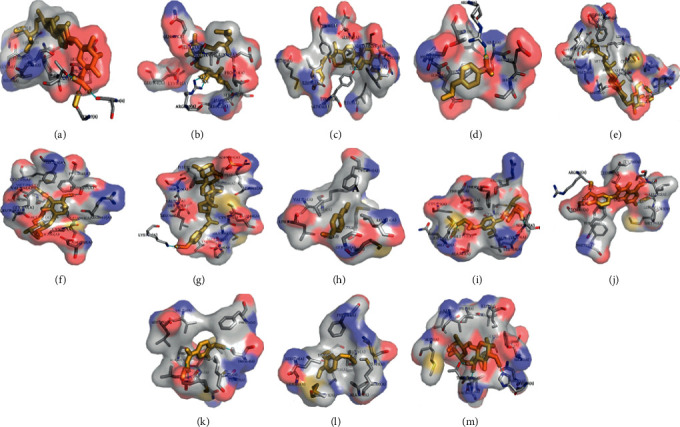
3D diagram of the optimal docking position of small molecule-proteins. *Note*. Best docking position of daucosterol and STAT3 (a); geranyl-1-O-*α*-L-arabinopyranosyl-(1 ⟶ 6)-*β*-D-glucopyranoside and STAT3 (b); 3*α*-O-(E)-p-hydroxy-cinnamoyl-olean-12-en-27-oic acid and STAT3 (c); anethole and STAT3 (d); daucosterol and EGFR (e); geranyl-1-O-*α*-L-arabinopyranosyl-(1 ⟶ 6)-*β*-D-glucopyranoside and EGFR (f); 3*α*-O-(E)-p-hydroxy-cinnamoyl-olean-12-en-27-oic acid and EGFR (g); anethole and EGFR (h); 3-methoxy-4-O-D-glucopyranosyl- phenylpropane-7, 8, 9-triol and EGFR (i); quercetin and EGFR (j); 3,4-dihydroxybenzoic acid and EGFR (k); benzene, 1, 2-dimethoxy-4-(1-propenyl)- and EGFR (l); geranyl-1-O-*α*-L-arabinopyranosyl-(1 ⟶ 6)-*β*-D-glucopyranoside and SLC10A2 (m).

Three proteins were involved in molecular docking including signal transducer and activator of transcription 3, STAT3 (6NJS), epidermal growth factor receptor, EGFR (5UG9), and ileal sodium/bile acid cotransporter, ASBT (3ZUY). Although the 3D structure of the three macromolecules including sodium/glucose cotransporter 1 (gene name SLC5A1), M-OR-1 (gene name OPRM1), and ileal sodium/bile acid cotransporter (gene name SLC10A2) from *Homo sapiens* could not be found in PDB, and bile acid metabolism is associated with diarrhea [24], we selected the protein of apical sodium-dependent bile acid transporter (ASBT) to replace SLC10A2, because it is used to simulate the 3D construction of protein encoded by SLC10A2 from *Homo sapiens* [25]. The grid boxes of the above macromolecules were defined and the configuration parameters were shown in Table S2. The 10 components of YT participated in molecular docking including daucosterol, anethole, quercetin, 3, 4-dihydroxybenzoic acid, benzene, geranyl-1-O-*α*-L-arabinopyranosyl-(1 ⟶ 6)-*β*-D-glucopyranoside, geranyl-1-O-*β*-D-xylopyranosyl-(1 ⟶ 6)-*β*-D-glucopyranoside, 3*α*-O-(E)-p-hydroxy-cinnamoyl-olean-12-en-27-oic acid, 3-methoxy-4-O-D-glucopyranosyl- phenylpropane-7, 8, 9-triol, and arbutin. Daucosterol also constituted three hydrogen bonds with EGFR residue including two oxygen atoms of carboxyl group on the *γ*-carbon atom of Glu (position 804, bond distances were 3.00 Å, 2.71 Å, and 2.78 Å, respectively). Furthermore, the affinity value on an optimal docking conformation between daucosterol and EGFR was −9.1 kcal/mol. Daucosterol formed three hydrogen bonds with STAT3 residue, which included Ser (position 614, bond distance = 3.07 Å), Gly (position 617, bond distance = 3.03 Å), and Thr (position 641, bond distance = 3.11 Å), and the affinity value on an optimal docking conformation between daucosterol and STAT3 was −7.5 kcal/mol. The affinity of the optimal docking result is shown in Table 4, and the optimal docking position for the above molecule with proteins is shown in Figure 8.

### 3.5. Mechanism Analysis of SCZX

According to the results of network pharmacology, SLC10A2, SLC5A1, EGFR, and STAT3 were defined as the core target proteins of YT-treating diarrhea, which were derived from the cluster analysis, involving two antidiarrheal modules. These core proteins could be mapped to 11 pathways, including cytokine–cytokine receptor interaction (hsa04060), Ras signaling pathway (hsa04014), Jak-STAT signaling pathway(hsa04630), MAPK signaling pathway (hsa04010), phospholipase D signaling pathway (hsa04072), HIF-1 signaling pathway (hsa04066), chemokine signaling pathway (hsa04062), bile secretion (hsa04976), ErbB signaling pathway (hsa04012), Th17 cell differentiation (hsa04659), and regulation of actin cytoskeleton (hsa04810). Most of them had been confirmed to be related to diarrhea, their functions and roles were identified, and their signaling pathways were closely related to intestinal inflammation, intestinal mucosal barrier, and water and ion transport. The details would be discussed later. Furthermore, molecular docking was provided to excavate the potential effective components of YT, and that included quercetin, geranyl-1-O- *α* -L-arabinopyranosyl-(1 ⟶ 6)- *β* -D-glucopyranoside, 3-methoxy-4-O-D-glucopyranosyl- phenylpropane-7, 8, 9-triol, 3*α*-O-(E)-p-hydroxy-cinnamoyl-olean-12-en-27-oic acid, and daucosterol. As one of the components of SCZX, BN is a clay comprising smectite minerals, mainly montmorillonite, which is a member of the smectite family with a general composition of (Na,Ca)_0.33_(Al,Mg)_2_(Si_4_O_10_) (OH)2nH_2_O [26]. It was reported that montmorillonite combined with zinc in diet could improve growth, alleviate postweaning diarrhea, and enhance intestinal mucosal integrity and digestive enzyme activities in the pancreatic and small intestinal contents of pigs [27].

**Figure 9:**
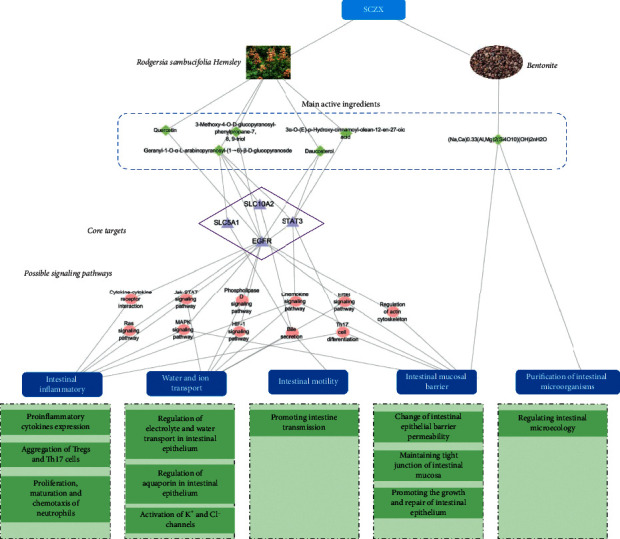
Potential active ingredients, mechanisms, and pharmacodynamic characteristics of SCZX.

Thus, we constructed a visual “herbal medicine-compound-target-pathway” network to explain the antidiarrheal mechanism of SCZX, which includes the potential active ingredients, core target proteins, and their pathways. And the pharmacological characteristics of SCZX were also linked to the network, which were summarized as the intestinal inflammatory, water and ion transport, intestinal motility, intestinal mucosal barrier, and purification of intestinal microorganisms. They are shown in Figure 9.

## 4. Discussions

The Yi people are mainly present in the Yunnan, Sichuan, Guizhou, and Guangxi Provinces in China, and traditional Yi medicine (TYM) has a long history of more than 1000 years and is a type of traditional Chinese medicine. The written history of TYM can be traced back to the *Yuanyang Yi Medicine Book*, written by ancient Yi authors in 957 AD [28] and was found in the Yuanyang County of Yunnan Province in 1985. The compatibility of principles of SCZX follows the theory of Ai and Bu based on TYM theory. As recorded in *Chinese Herbal Medicine of Yunnan* (Yunnan Health Bureau, Yunnan People's Publishing House 1971), the efficacy of BN is in its astringency to stop diarrhea, and the efficacy of YT is in its capacity for expelling wind-dampness and stopping diarrhea. YT has been used to treat diarrhea, abdominal distension, and dyspepsia among the Yi people, and the Yantuo Zhixie decoction is a folk prescription in TYM that only contains YT [29] and can be used to treat diarrhea. SCZX could reduce diarrhea via astringency and strengthening of the spleen and stomach in Yi medicine theory, which is often used clinically to treat autumn diarrhea, acute diarrhea, and persistent diarrhea in children [7].

Sennae Folium is a laxative and a traditional Chinese Materia Medica (CMM) that can cause diarrhea as well, commonly used for the treatment of diet, stress, or medication-related constipation, and it is very often used to construct animal models to support modern research on diarrhea-related diseases. The anthranoids are considered as the major contributor to diarrhea caused by Sennae Folium [30]. In our study, Sennae Folium was used to establish an acute diarrhea model in mice, and in model mice, diarrhea appeared after 2 times of administration of Folium Senna; and SCZX could reduce the diarrhea rate (DR), loose stool rate (LSR), loose stool level (LSL), and loose stool index (LSI) of the diarrhea mice. Small intestine propulsion rate also showed a tendency to decrease in groups receiving SCZX, indicating that SCZX may possess antidiarrheal activity.

Network pharmacology is a novel approach for investigating the system-level mechanisms of drugs, which was put forward by Hopkins in 2007 [31, 32]. Network pharmacology can build networks to reflect and clarify the interactive relationship between multiple components, multiple targets, multiple pathways of CMM, and complex diseases [11]. Recently, network pharmacology has become a novel method to investigate the system-level mechanisms of herbs or herbal formulae, highlighting the potential of traditional herbal medicine in “multicompound, multitarget” therapeutics [33]. In SCZX, YT belongs to a herbal medicine, but the pharmaceutical research on YT is still insufficient. Thus, in the study, we used network pharmacology, combined with molecular docking, to explain the antidiarrheal mechanism of YT and predict its effective ingredients.

In our study, considering the diversity of antidiarrheal approaches, the DL and ADME properties of YT's components were not screened before constructing the compound-target relationship. Primarily, all of the components were included for target prediction, and the core targets of YT in the treatment of diarrhea originated from the common targets of the gene-diarrhea association data of YT and diarrhea disease targets. The gene-diarrhea association data of YT were enriched through DisGeNET, which is integrated in the Enrichr tools. DisGeNET integrates data from CTD (Comparative Toxicogenomics Database), UniProt, OMIM, ClinVar, Orphanet, GWAS Catalog, GAD (Genetic Association Database), and LHGDN (The Literature Human Gene Derived Network) data resources, and it can also provide the Disease Specificity Index (DSI) and Disease Pleiotropy Index (DPI) to assist the prioritization of genotype-phenotype relationships. DisGeNET can be used to research the molecular underpinnings of specific human diseases and their comorbidities, analyze the properties of disease genes, and predict drug therapeutic action and drug adverse effects [17]. Relying on DisGeNET, we obtained 79 target proteins of YT diarrhea; furthermore, the core targets were defined as the key functional proteins of YT in the treatment of diarrhea. Then, using MCODE, the antidiarrheal effects of YT were focused on two functional clusters (CNA and CNB), screened out according to the distribution of these core proteins in the network. Finally, we performed GO analysis and signal pathway enrichment of these targets.

As determined by network pharmacology research, the mechanism of YT for treating diarrhea may involve the targets of STAT3, EGFR, SLC5A1, and SLC10A2. STAT3 is one of the major transcriptional regulators of inflammatory signaling. The inflammatory response triggered by the activation of STAT3 can cause dysregulation of the intestinal epithelial barrier [34] and intestinal inflammation associated with dysregulated electrolyte and water transport and result in diarrhea. Thus, it will be beneficial to reduce intestinal inflammation by effectively inhibiting STAT3 activation and the subsequent expression of inflammatory mediators. Interferon-*γ* may contribute to diarrhea associated with intestinal inflammation in part through the regulation of epithelial aquaporin-1 water channel via a nonclassical Jak-STAT signaling pathway [35]. Th17 cells are differentiated from CD4^+^ T cells stimulated by antigen and maintain their mucosal barrier function in the normal intestine, while Th17 cells can secret inflammatory factors such as IL-17A, IL-17B, IL-17C, IL-17D, IL-17E, and IL-17F, which mediate tissue inflammatory response by inducing the expression of proinflammatory cytokines, inflammatory chemokines, and matrix metalloproteinases. They also participate in the proliferation, maturation, and chemotaxis of neutrophils [36, 37], thus indicating that Th17 cells play an important role in the induction and maintenance of chronic intestinal inflammatory response. Intestinal mucositis caused an accumulation of Tregs and Th17 cells, which can exacerbate intestinal damage, diarrhea, neutrophil infiltration, and animal mortality [38].

EGF is an endogenous substance for tissue repair and cell protection secreted by the salivary glands, liver, pancreas, kidney, and intestines. EGF exhibits a wide range of physiological effects including the stimulation of electrolyte and nutrient absorption and the protective effects of gastrointestinal mucosa [39]. EGF mediates its effects via binding to its receptor, EGFR, which includes four subtypes of ErB1, ErB2, ErB3, and ErB4, and is expressed on the basolateral surface in human intestinal epithelial cells. EGFR-related signaling pathways contain RAS/MAPK, NF-*κ*B, PI3K/AKT/mTOR, and Jak/STAT [40]. ErbB receptor tyrosine kinase inhibitors (EGFR-TKIs), often used in cancer chemotherapy, can inhibit EGFR signaling in intestinal epithelial cells and decrease the capacity for growth and repair of the intestinal epithelium, which will result in atrophy of intestinal mucosa and secretory diarrhea [41]. Diarrhea is widespread among intestinal diseases involving ischemia and/or hypoxia, and HIF-1 can control epithelial ion and water transport in the intestinal mucosa, which mediates the repression of cystic fibrosis transmembrane conductance regulator in the intestinal epithelium [42]. Furthermore, phospholipase D signaling pathway [43], MAPK and Ras signaling pathways, cytokine–cytokine receptor interaction pathway [44, 45], regulation of actin cytoskeleton [46], and intestinal bile acid malabsorption [47] are related to the occurrence of diarrhea. Therefore, the antidiarrheal mechanism of YT may be related to the abovementioned proteins and possibly to their signal pathways.

Molecular docking was used to explore the effective ingredients of YT for diarrhea, and these compounds which conformed to IA, OB, and DL were selected out to perform docking. If the affinity is less than -7.0 kcal/mol, it means that small molecule has better binding activity with protein [48]. The results indicated that the 4 potential active components such as quercetin, geranyl-1-O-*α*-L-arabinopyranosyl-(1 ⟶ 6)-*β*-D-glucopyranoside, 3*α*-O-(E)-p-hydroxy-cinnamoyl-olean-12-en-27-oic acid, and daucosterol showed fine docking activities with the macromolecules of STAT3, EGFR, and SLC10A2.

## 5. Conclusion

Herbal approaches are being preferred for clinically treating diarrhea in children due to the adverse effects of chemical drugs, and the vast availability of antidiarrheal traditional medicine has shown efficacy. In this study, SCZX as an ancient prescription of Yi medicine was further verified to treat acute diarrhea. Depending on the focus study of YT via network pharmacology and molecule docking, we outlined the characteristics of antidiarrheal effect and “multicompounds, multitargets” mechanisms of SCZX completely, which include five aspects: inhibiting intestinal inflammation, acting as a barrier to the intestinal mucosal, regulating water and ion transport, involving the purification of intestinal microorganisms, and intestinal transmission. The results of this study might provide a clear direction for subsequent studies, but the extensive related experiments will be the focus of our next work.

## Figures and Tables

**Table 1:** Classification of stools.

Level	1	2	3	4
Stain diameter (cm)	<1	1–1.9	2–3	>3

**Table 2:** KEGG pathways of YT in the treatment of diarrhea.

No.	Term	Description	Gene symbols	Attribution
1	hsa04659	Th17 cell differentiation	IKBKB, IL2, STAT1, **STAT3**	CNA
2	hsa04062	Chemokine signaling pathway	IKBKB, CXCL8, STAT1, **STAT3**	CNA
3	hsa04630	Jak-STAT signaling pathway	IL2, STAT1, **STAT3**	CNA
4	hsa04068	FoxO signaling pathway	AKT1, **EGFR**, IL6, NRAS, PIK3R1	CNB
5	hsa04014	Ras signaling pathway	AKT1, **EGFR**, KIT, NRAS, PIK3R1, VEGFA	CNB
6	hsa04066	HIF-1 signaling pathway	AKT1, **EGFR**, IL6, PIK3R1, VEGFA	CNB
7	hsa04072	Phospholipase D signaling pathway	AKT1, **EGFR**, KIT, NRAS, PIK3R1	CNB
8	hsa04012	ErbB signaling pathway	AKT1, **EGFR**, NRAS, PIK3R1	CNB
9	hsa04510	Focal adhesion	AKT1, **EGFR**, PIK3R1, VEGFA	CNB
10	hsa04010	MAPK signaling pathway	AKT1, **EGFR**, NRAS, TNF	CNB
11	hsa04810	Regulation of actin cytoskeleton	**EGFR**, NRAS, PIK3R1	CNB
12	hsa04060	Cytokine-cytokine receptor interaction	**EGFR**, IL6, KIT, TNF, VEGFA, TYMS, ACE	CNB
13	hsa04976	Bile secretion	CYP3A4, **SLC5A1**, **SLC10A2**, ABCB11	Unclustered

**Table 3:** Information of active compounds and potential targets.

Id	Compounds	IA	OB	DL	Gene symbol
YT01	Daucosterol	+	−	+	STAT3, EGFR, OPRM1, KIT, TNF, SLC, CYP3A4
YT02	(E)-3, 7-Dimethyl-1-O-[*α*-L-rhamnopyranosyl-(1 ⟶ 6)-*β*-D-glucopyranosyl]-oct-2-en-7-ol	−	−	−	STAT3, TNF, BTK, SLC5A1
YT03	7-Dimethyl-1-O-[*α*-L-arabinofuranosyl-(1 ⟶ 6)-*β*-D-glucopyranosyl]-oct-2-en-7-ol	−	−	−	STAT3, EGFR, VEGFA, TNF, LCK, BTK, ACE, SLC5A1
YT04	Geranyl-1-O-*α*-L-arabinopyranosyl-(1 ⟶ 6)-*β*-D-glucopyranoside	−	−	+	STAT3, EGFR, VEGFA, KIT, TNF, LCK, BTK, ACE, SLC5A1, SLC10A2
YT05	Geranyl-1-O-*β*-D-xylopyranosyl-(1 ⟶ 6)-*β*-D-glucopyranoside	−	−	+	STAT3, EGFR, VEGFA, KIT, TNF, ACE, SLC5A1, SLC10A2
YT06	3*α*-O-(E)-p-Hydroxy-cinnamoyl-olean-12-en-27-oic acid	−	−	+	STAT3, EGFR, VEGFA, KIT, TNF, LCK, BTK, ACE, SLC5A1
YT07	3-O-Galloyl-epicatechin	−	−	+	STAT3
YT08	1-O-Galloyl-*β*-D-glucose	−	−	+	STAT3, OPRM1, VEGFA, ACE, VDR, ABCB11
YT09	Anethole	+	+	+	STAT3, EGFR, JAK3, ACE
YT10	Myristic acid	+	−	−	VDR
YT11	3-Methoxy-4-O-D-glucopyranosyl- phenylpropane-7, 8, 9-triol	−	−	+	EGFR, LCK, ACE, SLC5A1
YT12	Bergenin	+	−	+	EGFR
YT13	4-O-Galloylbergenin	+	−	−	EGFR, NRAS, VEGFA
YT14	11-O-Galloylbergenin	+	−	−	EGFR, NRAS, VEGFA
YT15	Quercetin	+	−	+	EGFR, PIK3R1
YT16	3, 4-Dihydroxybenzoic acid	+	+	+	EGFR, OPRM1, LCK
YT17	Benzene, 1, 2-dimethoxy-4-(1-propenyl)-	+	+	+	EGFR, JAK3
YT18	Rutin	+	−	−	OPRM1
YT19	Ethyl gallate	+	+	+	ADAM17
YT20	Ergosterol	+	−	+	JAK3, OPRM1, VDR
YT21	Geranyl 6-O-*β*-D-xylopyranosyl-*β*-D-glucopyranoside	−	−	+	SLC5A1
YT22	Carvacrol	+	+	+	JAK3
YT23	3, 3′, 4′, 5, 7-pentahydroxyflavan	+	−	−	VEGFA, KIT, LCK
YT24	3-O-*β*-Hydroxy-*δ*-8-*β*-(3, 4-dihydroxyphenyl)-pentanone	+	−	+	VEGFA, ACE
YT25	Eugenol	+	−	+	VEGFA, JAK3
YT26	*β*-Sitosterol	+	−	+	TNF, VDR, CYP3A4
YT27	6-O-Galloyl-D-glucose	+	−	+	TNF, ACE
YT28	Oleanolic acid	+	+	+	TNF
YT29	3, 5-Dimethoxy-4-O-*β*-D-glucopyranosyl-phenylpropane-7, 8, 9-Triol	−	−	−	LCK, BTK, ACE, SLC5A1
YT30	Arbutin	−	−	+	LCK, SLC5A1
YT31	Stearic acid	−	−	+	ACE, VDR
YT32	Citronellal	+	+	+	ACE
YT33	Hexadecanoic acid	+	−	−	VDR
YT34	(−)-limonene	+	+	+	VDR
YT35	Lauric acid	+	−	+	VDR
YT36	Geranyl-1-O-*α*-L-rhamnopyranosyl-(1 ⟶ 6)-*β*-D-glucopyranoside	−	−	+	TNF, **STAT3**, EGFR, VEGFA, NRAS, IL2, KIT, ADAM17, ACE

*Note*. OB : human oral bioavailability; IA : human intestinal absorption; DL : drug-likeness.

**Table 4:** Affinity of optimal docking conformation of the component and macromolecule.

Gene name	PDB ID	Compound	Affinity (kcal/mol)
STAT3	6NJS	Daucosterol	−7.5
STAT3	6NJS	Anethole	−4.5
STAT3	6NJS	Geranyl-1-O-*α*-L-arabinopyranosyl-(1 ⟶ 6)-*β*-D-glucopyranoside	−7.0
STAT3	6NJS	3*α*-O-(E)-p-Hydroxy-cinnamoyl-olean-12-en-27-oic acid	−8.1
EGFR	5UG9	Daucosterol	−9.1
EGFR	5UG9	Anethole	−6.1
EGFR	5UG9	Quercetin	−8.7
EGFR	5UG9	3,4-Dihydroxybenzoic acid	−5.9
EGFR	5UG9	Benzene, 1, 2-dimethoxy-4-(1-propenyl)-	−6.7
EGFR	5UG9	Geranyl-1-O-*α*-L-arabinopyranosyl-(1 ⟶ 6)-*β*-D-glucopyranoside	−8.4
EGFR	5UG9	3*α*-O-(E)-p-Hydroxy-cinnamoyl-olean-12-en-27-oic acid	−9.1
EGFR	5UG9	3-Methoxy-4-O-D-glucopyranosyl- phenylpropane-7, 8, 9-triol	−7.0
SLC10A2	3ZUY	Geranyl-1-O-*α*-L-arabinopyranosyl-(1 ⟶ 6)-*β*-D-glucopyranoside	−9.2

## Data Availability

The data used to support the findings of this study are available from the first author upon request.

## Abbreviations

WHO: World Health Organization
SCZX: Sechang-Zhixie-San
BN: Bentonite
YT: *Rodgersia sambucifolia* Hemsley (Yangtuo)
TCM: Traditional Chinese medicine
LSL: Loose stool level
DR: Diarrhea rate
LSR: Loose stool rate
LSI: Loose stool index
PPI: Protein–protein interactions
OMIM: Online Mendelian Inheritance in Man
GO: Gene ontology
ADME: Absorption, distribution, metabolism, and excretion
DL: Drug-likeness
OB: Human oral bioavailability
IA: Intestinal absorption
PDB: Protein data bank
CNA: Cluster networks A
CNB: Cluster networks B
BP: Biological process
CC: Cellular component
MF: Molecular function
OB: Human oral bioavailability
IA: Intestinal absorption
ASBT: Apical sodium-dependent bile acid transporter
TYM: Traditional Yi medicine
GAD: Genetic association database
LHGDN: The literature human gene derived network
DSI: Disease specificity index
DPI: Disease pleiotropy index
EGFR-TKIs: ErbB receptor tyrosine kinase inhibitors
KEGG: Kyoto encyclopedia of genes and genomes
CMM: Chinese materia medica.

## Supplementary Materials

Supplementary Materials Table S1: topological analysis of targets related to YT. Table S2: the definition of macromolecule grid box.
