# Supplementary material for: Antidiarrheal Effect of Sechang-Zhixie-San on Acute Diarrhea Mice and Network Pharmacology Deciphering Its Characteristics and Potential Mechanisms
Source: Evid Based Complement Alternat Med. 2020 Dec 11;2020:8880298. doi: 10.1155/2020/8880298 (PMC7749774; doi:10.1155/2020/8880298)
Supplement: Supplementary Materials — Table S1: topological analysis of targets related to YT. Table S2: the definition of macromolecule grid box. [file 8880298.f1.zip › 8880298.f1/table S1 (1).docx]

**Table S1 Topological analysis of targets related with YT**

| Gene name | MCODE_Cluster | Degree | Betweenness Centrality | Closeness Centrality | Average Shortest PathLength | MCODE_Score |
| --- | --- | --- | --- | --- | --- | --- |
| IKBKB | Cluster 1 | 11 | 0.00219018 | 0.46060606 | 2.17105263 | 9 |
| PLG | Cluster 1 | 14 | 0.00186262 | 0.50331126 | 1.98684211 | 10 |
| SERPINE1 | Cluster 1 | 15 | 0.00277833 | 0.5 | 2 | 10 |
| STAT3 | Cluster 1 | 34 | 0.05199088 | 0.61290323 | 1.63157895 | 8.935064935 |
| PTPRC | Cluster 1 | 22 | 0.00723447 | 0.54285714 | 1.84210526 | 9.808333333 |
| ERBB2 | Cluster 1 | 28 | 0.02180583 | 0.58015267 | 1.72368421 | 10.85897436 |
| STAT1 | Cluster 1 | 20 | 0.0033439 | 0.50666667 | 1.97368421 | 10.85897436 |
| CASP8 | Cluster 1 | 16 | 0.0051472 | 0.51006711 | 1.96052632 | 11 |
| CXCL8 | Cluster 1 | 28 | 0.02000887 | 0.5984252 | 1.67105263 | 9.274509804 |
| NOS2 | Cluster 1 | 15 | 0.01220529 | 0.50666667 | 1.97368421 | 9.848484848 |
| IL2 | Cluster 1 | 26 | 0.01490978 | 0.56716418 | 1.76315789 | 9.544117647 |
| PTGS2 | Cluster 1 | 22 | 0.01723049 | 0.55072464 | 1.81578947 | 9.274509804 |
| ADAM17 | Cluster 1 | 11 | 5.37E-04 | 0.47798742 | 2.09210526 | 9 |
| HPGDS | Cluster 1 | 22 | 0.04360639 | 0.56716418 | 1.76315789 | 9 |
| MPO | Cluster 1 | 22 | 0.0310369 | 0.55072464 | 1.81578947 | 9.120879121 |
| ABCB1 | Cluster 1 | 27 | 0.05679289 | 0.59375 | 1.68421053 | 9 |
| CTSB | Cluster 1 | 13 | 0.00277386 | 0.47204969 | 2.11842105 | 9 |
| TYMS | Cluster 2 | 15 | 0.04747282 | 0.52054795 | 1.92105263 | 7 |
| TNF | Cluster 2 | 34 | 0.0420501 | 0.62295082 | 1.60526316 | 7.652173913 |
| EGFR | Cluster 2 | 38 | 0.09960459 | 0.64957265 | 1.53947368 | 7.565217391 |
| IL6 | Cluster 2 | 43 | 0.09586869 | 0.67256637 | 1.48684211 | 6.838624339 |
| NRAS | Cluster 2 | 15 | 0.00374441 | 0.475 | 2.10526316 | 7.03030303 |
| JAK3 | Cluster 2 | 14 | 8.00E-04 | 0.48101266 | 2.07894737 | 8.010989011 |
| PIK3R1 | Cluster 2 | 17 | 0.00670637 | 0.48717949 | 2.05263158 | 8.181818182 |
| VEGFA | Cluster 2 | 37 | 0.05203206 | 0.64957265 | 1.53947368 | 7.652173913 |
| LCK | Cluster 2 | 15 | 0.00352029 | 0.48101266 | 2.07894737 | 7.542857143 |
| AKT1 | Cluster 2 | 40 | 0.0921177 | 0.66666667 | 1.5 | 6.988603989 |
| KIT | Cluster 2 | 20 | 0.00757989 | 0.52777778 | 1.89473684 | 7.014705882 |
| OPRM1 | Cluster 2 | 16 | 0.04820654 | 0.5170068 | 1.93421053 | 7 |
| LGALS3 | Cluster 2 | 15 | 0.0032904 | 0.50666667 | 1.97368421 | 7.151515152 |
| BTK | Cluster 2 | 10 | 3.50E-04 | 0.44186047 | 2.26315789 | 8 |
| ACE | Cluster 2 | 21 | 0.05100239 | 0.54676259 | 1.82894737 | 8.454545455 |
| VDR | Cluster 2 | 12 | 0.00433994 | 0.50331126 | 1.98684211 | 8 |
| UGT2B7 | Cluster 3 | 12 | 0.00602746 | 0.44970414 | 2.22368421 | 5.333333333 |
| NR1I2 | Cluster 3 | 16 | 0.01215699 | 0.52413793 | 1.90789474 | 6.222222222 |
| CES1 | Cluster 3 | 12 | 0.01262645 | 0.45783133 | 2.18421053 | 5.5 |
| CYP1A2 | Cluster 3 | 11 | 0.0197265 | 0.43930636 | 2.27631579 | 5.5 |
| CYP2A6 | Cluster 3 | 9 | 0.00316685 | 0.43181818 | 2.31578947 | 5.571428571 |
| CYP2D6 | Cluster 3 | 10 | 0.00262068 | 0.43678161 | 2.28947368 | 5.571428571 |
| TTR | Cluster 3 | 10 | 0.01519041 | 0.47798742 | 2.09210526 | 5 |
| GCG | Cluster 3 | 13 | 0.01990108 | 0.50331126 | 1.98684211 | 5.785714286 |
| ABCG2 | Cluster 3 | 22 | 0.04527378 | 0.56716418 | 1.76315789 | 5.833333333 |
| HNF4A | Cluster 3 | 14 | 0.00986766 | 0.5170068 | 1.93421053 | 6 |
| DHFR | Unclustered | 11 | 0.02030222 | 0.49032258 | 2.03947368 | 5 |
| MAP3K7 | Unclustered | 3 | 0 | 0.4021164 | 2.48684211 | 3 |
| TYMP | Unclustered | 9 | 0.01437691 | 0.475 | 2.10526316 | 1.928571429 |
| CDA | Unclustered | 5 | 0.00252366 | 0.3877551 | 2.57894737 | 3 |
| BRAF | Unclustered | 5 | 4.18E-04 | 0.43678161 | 2.28947368 | 4 |
| RAP1A | Unclustered | 6 | 0.00306827 | 0.42696629 | 2.34210526 | 2.4 |
| CYP3A4 | Unclustered | 21 | 0.03529303 | 0.54676259 | 1.82894737 | 4.769230769 |
| RET | Unclustered | 9 | 0.00149152 | 0.46341463 | 2.15789474 | 5.333333333 |
| CES2 | Unclustered | 8 | 0.00362091 | 0.41758242 | 2.39473684 | 4.761904762 |
| TOP2A | Unclustered | 6 | 0.00408265 | 0.45238095 | 2.21052632 | 3.733333333 |
| POLA1 | Unclustered | 4 | 5.82E-04 | 0.35514019 | 2.81578947 | 3 |
| LGALS1 | Unclustered | 5 | 2.92E-05 | 0.44186047 | 2.26315789 | 3.733333333 |
| ABL1 | Unclustered | 16 | 0.00344198 | 0.51006711 | 1.96052632 | 6.241758242 |
| CAPN2 | Unclustered | 5 | 2.43E-04 | 0.41758242 | 2.39473684 | 2.7 |
| GSTK1 | Unclustered | 2 | 8.85E-05 | 0.3857868 | 2.59210526 | 0.666666667 |
| HEXB | Unclustered | 4 | 0.00955325 | 0.4021164 | 2.48684211 | 2 |
| DGAT1 | Unclustered | 4 | 0.0026134 | 0.40425532 | 2.47368421 | 2 |
| MME | Unclustered | 13 | 0.00465391 | 0.49673203 | 2.01315789 | 5.166666667 |
| HTR3A | Unclustered | 5 | 0.00133339 | 0.40641711 | 2.46052632 | 2.7 |
| SLC6A4 | Unclustered | 7 | 0.00533215 | 0.4691358 | 2.13157895 | 2.4 |
| NR3C2 | Unclustered | 6 | 0.00442257 | 0.44444444 | 2.25 | 4 |
| MAPT | Unclustered | 7 | 0.00104123 | 0.45238095 | 2.21052632 | 3.238095238 |
| CYP11B2 | Unclustered | 2 | 0 | 0.35680751 | 2.80263158 | 2 |
| ADA | Unclustered | 9 | 0.0105189 | 0.48407643 | 2.06578947 | 4 |
| ABCB11 | Unclustered | 7 | 0.00145889 | 0.46341463 | 2.15789474 | 3 |
| SLC10A2 | Unclustered | 7 | 0.00589163 | 0.42222222 | 2.36842105 | 3.733333333 |
| POLG | Unclustered | 3 | 1.86E-04 | 0.35023041 | 2.85526316 | 1.666666667 |
| SLC5A1 | Unclustered | 5 | 0.02128101 | 0.42696629 | 2.34210526 | 1.666666667 |
| SI | Unclustered | 4 | 0.00831187 | 0.38190955 | 2.61842105 | 1.4 |
| TREH | Unclustered | 2 | 0 | 0.30894309 | 3.23684211 | 2 |
| FAAH | Unclustered | 4 | 0.00114551 | 0.4 | 2.5 | 2 |
| MPI | Unclustered | 2 | 5.72E-04 | 0.3220339 | 3.10526316 | 0.666666667 |
| SCN9A | Unclustered | 1 | 0 | 0.34234234 | 2.92105263 | 0 |
| DNMT3B | Unclustered | 2 | 1.95E-04 | 0.40860215 | 2.44736842 | 0.666666667 |
| DHFR | Unclustered | 11 | 0.02030222 | 0.49032258 | 2.03947368 | 5 |
| MAP3K7 | Unclustered | 3 | 0 | 0.4021164 | 2.48684211 | 3 |
| TYMP | Unclustered | 9 | 0.01437691 | 0.475 | 2.10526316 | 1.928571429 |
| CDA | Unclustered | 5 | 0.00252366 | 0.3877551 | 2.57894737 | 3 |
| BRAF | Unclustered | 5 | 4.18E-04 | 0.43678161 | 2.28947368 | 4 |
| RAP1A | Unclustered | 6 | 0.00306827 | 0.42696629 | 2.34210526 | 2.4 |
| CYP3A4 | Unclustered | 21 | 0.03529303 | 0.54676259 | 1.82894737 | 4.769230769 |
| RET | Unclustered | 9 | 0.00149152 | 0.46341463 | 2.15789474 | 5.333333333 |
| CES2 | Unclustered | 8 | 0.00362091 | 0.41758242 | 2.39473684 | 4.761904762 |
| TOP2A | Unclustered | 6 | 0.00408265 | 0.45238095 | 2.21052632 | 3.733333333 |
| POLA1 | Unclustered | 4 | 5.82E-04 | 0.35514019 | 2.81578947 | 3 |
| LGALS1 | Unclustered | 5 | 2.92E-05 | 0.44186047 | 2.26315789 | 3.733333333 |
| ABL1 | Unclustered | 16 | 0.00344198 | 0.51006711 | 1.96052632 | 6.241758242 |
| CAPN2 | Unclustered | 5 | 2.43E-04 | 0.41758242 | 2.39473684 | 2.7 |
| GSTK1 | Unclustered | 2 | 8.85E-05 | 0.3857868 | 2.59210526 | 0.666666667 |
| HEXB | Unclustered | 4 | 0.00955325 | 0.4021164 | 2.48684211 | 2 |
