# Supplementary material for: Antidiarrheal Effect of Sechang-Zhixie-San on Acute Diarrhea Mice and Network Pharmacology Deciphering Its Characteristics and Potential Mechanisms
Source: Evid Based Complement Alternat Med. 2020 Dec 11;2020:8880298. doi: 10.1155/2020/8880298 (PMC7749774; doi:10.1155/2020/8880298)
Supplement: Supplementary Materials — Table S1: topological analysis of targets related to YT. Table S2: the definition of macromolecule grid box. [file 8880298.f1.zip › 8880298.f1/table S2 (1).docx]

Table S2 the definition of macromolecule grid box

| PDB ID | x | y | z | spacing |
| --- | --- | --- | --- | --- |
| 6NJS | 34 | 34 | 34 | 1 |
| 5UG9 | 24 | 24 | 24 | 1 |
| SLC10A2 | 22 | 22 | 22 | 1 |
